# Identification of HPV Types and Mycobacterium Tuberculosis Complex in Historical Long-Term Preserved Formalin Fixed Tissues in Different Human Organs

**DOI:** 10.1371/journal.pone.0170353

**Published:** 2017-01-23

**Authors:** Maja Hühns, Andreas Erbersdobler, Annette Obliers, Paula Röpenack

**Affiliations:** Institute of Pathology, University Medicine of Rostock, Rostock, Germany; Hebrew University, ISRAEL

## Abstract

University anatomical-pathological collections represent huge sources of human tissues and preparations from a variety of different diseases. With the help of modern genetic and histological methods, preserved fixed tissues from pathological collections can be used to re-evaluate former diagnoses. We analysed 25 specimens from our pathological collection with ages ranging from 78 to 112 years. The tissues originated from the oral cavity, lip, tongue, lung, bone, kidney, spleen, thymus, larynx, lymph node, penis and uterine cervix with an original diagnosis of epithelial cancers or tuberculosis. Amplifiable DNA was extracted and in epithelial cancers, potential HPV infection was investigated. Specimens with an original diagnosis of tuberculosis were examined for mycobacterial infection. The tissues were also examined using modern histological methods. Our data showed that in 24/25 specimens the histological structure was preserved and in 10/11 specimens the diagnosis of squamous cell carcinoma could be confirmed. Additionally, HPV type 16 was detected in 8 specimens. The histological pattern of tuberculosis was found in 11/14 specimens and the *Mycobacterium tuberculosis* complex was ascertained in four specimens. Our study showed that pathogens such as HPV or *Mycobacterium tuberculosis* can be detected in historical pathological preparations, and that these collections are suitable for further epidemiological research.

## Introduction

University anatomical-pathological collections contain a huge quantity of human tissue and preparations from a variety of different diseases, some of which are uncommon today. Therefore, they are now of enormous value with regard to education and research. However, working with old pathological preparations raised several potential difficulties: First, since many pathological collections date from a time when most diagnoses were made macroscopically and without histology, it is questionable whether all original diagnoses comply with modern standards. Thus, it is important to verify the original diagnosis using modern histological methods before these preparations can be used for education or research. Second, these specimens have been preserved for many years in solutions such as ethanol or formalin which could have potentially changed the molecular structure. Therefore, it is unclear whether these old preparations are actually suitable for examination by modern laboratory methods.

In this proof-of-concept study, we investigated long-term preserved, formalin-fixed preparations via modern histological and molecular genetic methods. This was carried out using potentially human papillomavirus (HPV) associated cancer specimens, and tuberculosis (TB) infected samples. Both diseases have a major impact on modern human health. HPV infection is the most common infectious disease of the reproductive tract today [1]. Additionally, TB is responsible for approximately 1.5 million deaths and 9 million new infections annually worldwide [2]. Moreover, HPV and TB have accompanied mankind for a long time. For example, *Mycobacteria tuberculosis* (MTB), the pathogen that triggers TB in most cases, has been detected in 9,000 to 11,000-year-old human bodies [3–6]. Additionally, MTB was found in ancient skeletal and mummified material [7–17], and was first detected via PCR in archaeological bone material remains of the 14^th^-16^th^ century. Evidence of HPV type 18 was found in mummified tissue dating back to the 16th century [18]. However, no data is available on the detection of HPV or MTB in long-term preserved tissue from pathologic-anatomical collections so far [19]. In previous publications great efforts have been made to extract historical DNA from tissues. Nevertheless, most of them refer to formalin fixed and paraffin embedded (FFPE) tissues (e.g. [20–25]. Modifications to experimental protocols were performed in order to extract high amounts of amplifiable DNA from samples including altering the concentration of proteinase K [21], altering incubation temperature [22] or duration of digestion period [21,22,24]. Fixation protocols differ for long-term fixed tissue and FFPE tissues, for example with regard to formalin concentration and duration of fixation. Additionally, a wide range of variables that could influence the quality of the DNA (e.g. temperature, light exposure, use of buffered or unbuffered formalin or formalin concentration) are often unknown in historical collections. For the first time, amplifiable DNA up to 171 bp from more than 50 year old formalin-fixed tissues was extracted by [26]. Recently, analyses of up to 80 year old formalin fixed tissues (of different cancer types) revealed amplifiable DNA fragments up to 381 bp and mutations were detected in the BRAF and KRAS genes [27]. Furthermore, immunohistochemical expression of different proteins such as vimentin and GFAP could also be observed in these specimens.

In the present study, 25 preparations from our own pathological collection of potentially HPV-associated epithelial cancers and tuberculosis, with ages ranging from 78 to 112 years, were investigated to detect pathogens via molecular methods and to verify the original diagnosis histologically.

## Materials and Methods

### Ethics statement

All investigated specimens had been collected in an anonymous manner several decades ago therefore informed consent of the patients could not be obtained. Actually, any personal impairment of persons—be they alive or dead—can be excluded by the design of the study. The use of human tissue samples for this study was endorsed by the local ethics committee (ethics commission / university medicine of Rostock, Rostock, Germany: project number: A2015-0061).

### Origin of tissue

**Fig 1:**
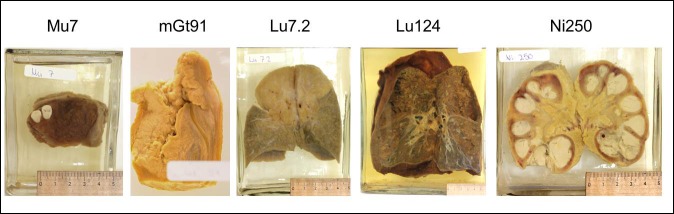
Wet preparations of 5 specimens stored in formalin and used for further investigations.

Tissue samples were obtained from human wet preparations stored at the University Pathological Collection Rostock. The following inclusion criteria were applied: (1) the original diagnosis was either tuberculosis or epithelial cancer of the oro-pharyngeal or genital region, (2) the disease was macroscopically visible, and (3) there were no signs of mould or autolysis. Accordingly, 25 preparations of oral cavity, lip, tongue, lung, bone, kidney, spleen, thymus, larynx, lymph node, penis and uterine cervix were chosen. Due to a lack of documentation, original fixation solution and long-term storage conditions were unrecorded for all preparations and the exact age could not be determined in most of the specimens. However, the year of fixation was registered for six specimens ranging from 1902 to 1937 (Table 1, Fig 1). Given the history of our collection, it is most likely that all preparations were approximately obtained in the first sixty years of the 20^th^ century (1900–1964) and either a formalin- or alcohol-based solution was used for fixation and preservation. Sampling and storage were performed in a standardized manner for every specimen as described [27].

**Table 1:** Overview of specimens, fixation time and their corresponding disease.

Specimen	organ	fixation time	original disease
Mu7	mouth cavity	1902	carcinoma of oropharynx
Mu10	mouth cavity	unknown	carcinoma of oropharynx
Mu17	lip	unknown	carcinoma of the lip
Mu28	lip	1937	carcinoma of the lip
Mu31	tongue	unknown	carcinoma of the tongue
Mu37	lip	unknown	carcinoma of the lip
Md40	mouth base	unknown	carcinoma of the base of the tongue
wGt151	cervix uteri	1932	cervical carcinoma
mGt26	penis	1924	penis carcinoma
mGt91	penis	1937	penis carcinoma
mGt103	penis	unknown	penis carcinoma
Lu7.2	lung	unknown	caseous pneumonia with local dissemination in a child
Lu30/10	lung	unknown	tuberculosis
Lu124	lung	1923	tuberculosis empyema
Lu145	lung	unknown	engraver lung /upper lobe tuberculosis
Lu180	lung	unknown	macronodular miliary tuberculosis
Lu217	lung	unknown	cavernous cirrhotic tuberculosis
Kn322	bone	unknown	extensive caseous tuberculosis of the backbone
Ni34	kidney	unknown	tuberculosis of the kidney
Ni250	kidney	unknown	caseous tuberculosis of the renal parenchyma
Mi133	spleen	unknown	miliary tuberculosis of the spleen
Thy3	thymus	unknown	tuberculosis of the thymus
Ke3	larynx	unknown	tuberculosis of the larynx
Gf122	lymph node	unknown	tuberculosis of the lymph node
wGt200	uteri	unknown	tuberculosis of the uterus and the vagina

### DNA extraction

Tumour DNA was extracted from 25 wet preparations. For each specimen 60 cyrosectioned slices (10 μm thick) were collected into 1.5 ml microtubes containing 400 μl tissue-lysis buffer (proteinase K (20mg/ml)) and digestion solution ((10mM Tris, 0.1mM EDTA, pH 8.0, and 0.5% Tween-20)). DNA was extracted as described in [27].

### Identification of HPV types

For identification of HPV types, the HPV Type 3.5 LCD-Array Kit (Chipron, Berlin, Germany) was used according to manufacturer’s instructions. Briefly, two PCR reactions were performed using amplified regions of the L1 gene. The My11/09 primers amplified a region of 450bp, as described in [28]. The ‘125’ primer (PCR product ca. 175bp) contained a mixture of type-specific oligonucleotides which is located within the My11/09 fragment. Subsequently, PCR products were analysed by agarose gel electrophoresis. Both PCR products were mixed and hybridized on the 3.5 LCD slide due to manufacturer’s instruction. Slides were subsequently scanned on the Slide Reader Scanner and evaluated using Slide Reader Software (Chipron, Berlin, Germany). All PCR amplifications and hybridisations on the chips were performed twice. Negative controls were performed in order to exclude false positive results.

### Identification of Mycobacteria spec. tuberculosis complex and other Mycobacteria

For identification of MTB complex and other Mycobacteria (MOTT) the Myco ^Direct^ 1.7 LCD-Array Kit (Chipron, Berlin, Germany) was used according to the manufacturer’s instructions. Briefly, two PCR reactions were performed based on the repetitive element IS6110 (product size 126bp) and rRNA spacer region (225-265bp) then analysed by agarose gel electrophoresis. Both PCR products were mixed and hybridized on the 1.7 LCD slide according to the manufacturer’s instructions. Slides were subsequently scanned on the Slide Reader Scanner and evaluated with the Slide Reader Software (Chipron, Berlin, Germany). All PCR amplifications and hybridisations on the chips were performed twice. Negative controls were performed to exclude false positive results.

### Treatment of DNA with uracil-DNA glycosylase (UDG)

To perform the UDG treatment, we added 1x UDG reaction buffer (New England BioLabs) and UDG (1 unit/reaction) directly to the DNA (subsequently used for specific PCR) and incubated for 30 minutes at 37°C.

### Determination of the *pncA* sequence

The *pncA* gene is involved in bacterial activation of pyrazinamide and has been used to differentiate *M*. *bovis* from other species of the MTB complex by a single point mutation [29]. At nucleotide position 169 of the *pncA* gene a change of C to G occurs in *M*. *bovis* causing the replacement of histidine (CAC) with aspartic acid (GAC) at amino acid position 57.

The sequence of the *pncA* gene was analysed in specimens positive for MTB. The Pyromark Assay Design 2.0 software (Qiagen) was used to design PCR primers and sequencing primers for the *pncA* mutation region. The primers for the Pyromark assay were as follows: forward primer: 5'-GCAACCAAGGACTTCCACATC-3', reverse primer: 5'- ACGAGGAATAGTCCGGTGTGC-3', sequencing primer: 5'-ATCGACCCGGGTGAC-3'. Briefly, 50ng of genomic DNA was used in 25μl reactions. Subsequently, 10μl of the PCR product was used for pyrosequencing with a Q24 Pyromark instrument (Qiagen, Hilden, Germany) according to the manufacturer’s instructions.

### Staining

For each case, 4 μm sections were cut from the appropriate paraffin block, transferred to an adhesive-coated glass slide system (Instrumedics Inc, Hackensack, NJ, USA) then stained with haematoxylin and eosin (H&E). All 13 cases with tuberculosis were additionally stained with the Ziehl-Neelsen stain and 10 cases were Gram stained.

## Results

### Extraction of DNA from wet preparations

We used 25 wet preparations, from specimens aged 78–112 years. Tumour DNA was successfully extracted from all 25 wet preparations using a protocol based on proteinase K [30] in moderate to high concentrations (Table 2). The total amount of DNA ranged from 0.14 to 22.63 μg (mean: 4.08μg). The purity of DNA, assessed by the ratio of absorbance at 260/280 nm was low in all samples (mean: 1.61) (Table 2).

**Table 2:** Comparison of total DNA amount and purity of analysed specimens.

Specimen	total yield (in μg DNA)	A_260_/A_280_
Mu7	1.94	1.53
Mu10	0.98	1.52
Mu17	6.22	1.53
Mu28	0.17	1.13
Mu31	3.3	1.49
Mu37	2.79	1.54
Md40	1.8	1.47
WGt151	2.19	1.12
mGt26	22.63	1.44
mGt91	5.98	1.56
mGt103	21.15	1.66
Lu7.2	4.13	1.5
Lu30/10	1.81	1.72
Lu124	4.43	1.7
Lu145	0.98	1.95
Lu180	4.1	1.56
Lu217	3.25	1.69
Kn322	1.41	1.7
Ni34	1.25	1.92
Ni250	0.14	2.15
Mi133	7.14	1.36
Thy3	1.79	1.75
Ke3	1.01	2.09
Gf122	1.09	1.95
wGt200	1.09	1.61

The median amount of DNA in the different organs ranged from 0.69±0.79 μg in kidney to 16.59±9.22 μg in penis (Table 3). The A_260_/A_280_ ratio ranged from 1.36 in spleen to 2.04±0.16 in kidney (Table 3).

**Table 3:** DNA concentration and DNA purity of the different investigated organs.

organ	median amount of DNA (in μg) and standard deviation	A_260_/A_280_ and standard deviation
mouth	2.46 ± 1.96	1.46 ± 0.14
lung	3.12 ± 1.42	1.69 ± 0.15
kidney	0.69 ± 0.79	2.04 ± 0.16
spleen	7.14	1.36
larynx	1.01	2.09
thymus	1.79	1.75
lymph node	1.09	1.95
uterus	1.64 ± 0.78	1.37 ± 0.34
penis	16.59 ± 9.22	1.55 ± 0.11

### Amplification and identification of HPV types and Mycobacteria species

**Fig 2:**
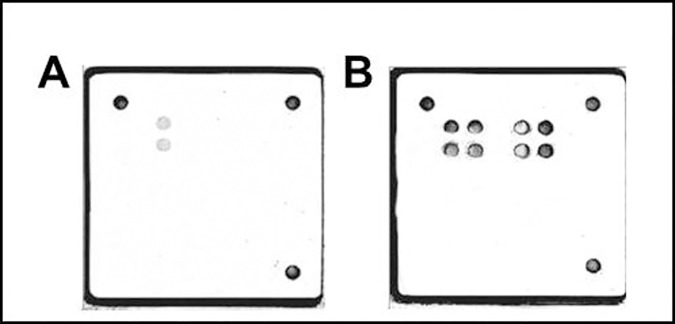
Detection of the pathogens by using the Chipron LCD Arrays. (A): Detection of HPV type 16 in mGt91. (B) Detection of *Mycobacterium* sp. in Lu124.

PCR amplification was performed targeting the 430 bp and 175 bp gene fragments for HPV typing. A positive amplification was detected in 10/11 specimens for HPV typing, but only the 175 bp fragment was amplifiable, which suffices for hybridization and is more sensitive for formalin fixed tissues (Table 4). All PCR reactions and subsequent hybridization assays were repeated. Additionally, several specimens were treated with UDG before PCR amplification. Again, our initial results were confirmed. Interestingly, HPV type 16 was detected in 8 PCR amplifiable specimens (Table 4, Fig 2A).

**Table 4:** Results of amplifiability and HPV typing.

specimen	amplification of My11/09	amplification of ‘125’	HPV type
Mu7	-^b^	+^a^	16 (weak)
Mu10	-	+	16 (weak)
Mu17	-	+	negative
Mu28	-	+	16
Mu31	-	-	
Mu37	-	+	negative
Md40	-	+	16
wGt151	-	+	16 (weak)
mGt26	-	+	16
mGt91	-	+	16
mGt103	-	+	16

^a^ (+) indicates positive amplification.

^b^ (-) indicates negative amplification.

For the identification of mycobacteria, the RNA spacer region (ranged from 225–265 bp) was amplifiable in 13/14 specimens and the IS6110 (126bp) could additionally be amplified in 4 specimens (Table 5). 13/14 specimens described as tuberculosis were positive for infection with the MTB complex, the *Mycobacterium avium* complex (MAC) or other MOTT (Table 5). We found 4 specimens (Lu124, Lu145, Lu217 and Gf122) infected with MTB, whereas Lu145 and Gf122 were additionally infected with MOTT (Table 5, Fig 2B). In 8 specimens only MOTT (not further classified by chip) were detected. MAC was identified in Ke3. All PCR reactions and subsequent hybridization assays were repeated, leading to the same results. Additionally, several specimens were treated with UDG before PCR amplification. Again, our initial results were confirmed.

**Table 5:** Results of amplifiability and identification of Mycobacteria.

Specimen	rRNA spacer region amplification (225–265 bp)	IS6110 amplification (126bp)	identified Myco-bacteria species
Lu7.2	+^a^	-	MOTT^b^
Lu30/10	+	-	MOTT
Lu124	+	+	MTB^c^
Lu145	+	+	MTB, MOTT
Lu180	+	-	MOTT
Lu217	+	+	MTB
Kn322	+	-	MOTT
Ni34	-	-	
Ni250	+	-	MOTT
Mi133	+	-	MOTT
Thy3	+	-	MOTT
Ke3	+	-	MAC^d^
Gf122	+	+	MTB, MOTT
wGt200	+	-	MOTT

^a^ (+) indicates positive amplification.

^b^ MOTT, mycobacteria other tuberculosis.

^c^ MTB; *Mycobacteria tuberculosis complex*.

^d^ MAC, *Mycobacteria avium complex*.

For the differentiation between *M*. *tuberculosis* and *M*. *bovis* the sequence of the *pncA* gene was determined in specimen Lu124, Lu145, Lu217 and Gf122. In Pyromark analyses the non-mutated form of the *pncA* sequence was detected in Lu124, Lu217 and Gf122, in Lu145 no amplification was possible.

### Morphological evaluation (haematoxylin-eosin stains)

**Fig 3:**
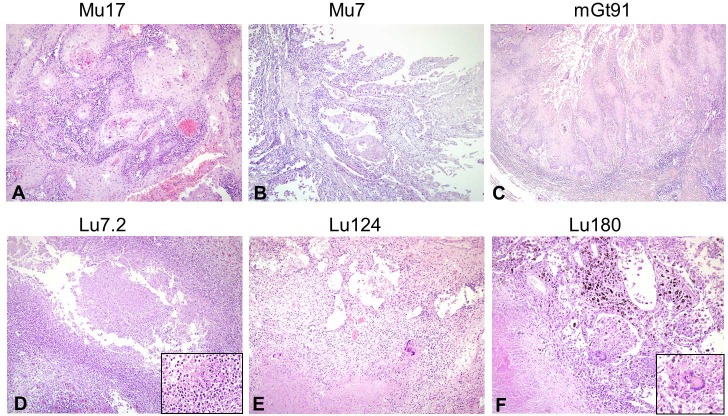
Newly prepared histological pictures of the old wet preparations. H&E stain of HPV-associated specimens (A-C) showed squamous cell carcinoma. In tuberculosis associated specimens (D-F) caseating necrosis, granulomas and Langerhans giant cells were found. A-F by 10x magnification; insert in D x40 magnification; insert in F x20 magnification.

For every specimen, a H&E stained histological slide was microscopically assessed by a pathologist (Fig 3). The histological structure was well preserved in 24/25 slides so that a reasonable histological assessment was possible in nearly all specimens. Only in specimen wGt200 (uterine tuberculosis) was the microscopic structure too badly preserved to allow a verification of the diagnosis. The specimens taken from squamous-cell carcinomas suspicious for HPV infection were assessed for typical neoplastic structures and tumour cells (Fig 3A–3C). In specimen wGt151 no tumour cells were found, whereas in the other 10/11 specimens the diagnosis of squamous cell carcinoma could be confirmed. The characteristic histological pattern of tuberculosis with signs of inflammation, necrosis and caseating granulomas (Fig 3D–3F) could be observed in 11/14 specimens. As mentioned above, specimen wGt200 showed no preserved microscopic structure at all, so that a definitive diagnosis could not be made. In addition, two specimens (Lu30/10 and Kn322) of lung and bone tissue respectively showed no particular sign of tuberculosis. In Lu30/10 massive blood congestion could be detected, whereas Kn322 showed a rather unspecific chronic inflammatory process.

### Analysis of tissues by special staining

All cases with tuberculosis were stained with Ziehl-Neelsen stain, but no acid-resistant rod-shaped bacteria were found. Furthermore, in all 10 cases stained with Gram stain no bacteria could be seen.

## Discussion

In this study, we investigated 25 specimens of TB and HPV-related cancer to determine whether the microscopic structure was sufficiently preserved to allow histological evaluation and whether molecular detection of respective pathogens was still feasible in long-term preserved tissue.

Apart from the specimen wGt200, the quality of the preserved microscopic structure was such that it allowed a reasonable histological diagnosis for every specimen. Thus, the original diagnosis could be confirmed in 10/11 cancer specimens and in 11/14 preparations originally labelled as TB. The four remaining specimens were either too badly preserved to make a diagnosis at all (wGt200) or showed no histological evidence of epithelial cancer (wGt151) or TB (Kn322 and Lu30/10). On the other hand, their microscopic appearances gave no specific indications for other diseases that might explain the macroscopic appearance. Therefore, they may reveal a sampling error, a common problem when working with archival specimens. These specimens were made to be presented to an interested audience and therefore biopsies were aimed to be as small as possible. However, taking only small biopsies bears the risk of getting tissue that is not representative for the actual disease. Therefore, it is possible that we simply biopsied an unrepresentative side of the specimen. This assumption is supported by the fact that the macroscopic picture of these three specimens matches with the original diagnosis and no other obvious microscopic diagnosis could be made. Unfortunately, we were not able to obtain new biopsies in these cases and thus cannot provide complete clarification regarding the real diagnosis of these specimens.

Special bacterial staining methods such as Ziehl-Neelsen or Gram revealed only negative results. Both staining methods require complete integrity of the bacterial cell wall, which may have been destroyed by formalin due to the longer fixation time. This effect of formalin can also be used for disinfection purposes even against the more resistant mycobacteria [31,32]. Hence, tissue that has been preserved in formalin appears to be unsuitable for the preservation of bacterial stains.

In the second part of the study, we performed molecular detection of either HPV or mycobacteria. We were able to obtain an amount of DNA of 4.08 μg (mean) from every specimen, which was similar to our recent published results [27]. The rather low A_260_/A_280_ ratio indicated protein contamination. This agrees with former studies showing the purity of DNA of old preparations extracted by proteinase K digestion to be beyond the optimal range of 1.8–2.0 [26,27].

It was possible to amplify sequences between 125 and 265 bp in 23/25 specimens. For HPV analyses, the 450 bp fragment (My11/09) was not amplifiable, most likely due to the fragmentation of the historical DNA. The 175 bp fragment (‘125’) of the L1 gene is located within the My11/09 fragment and more sensitive for formalin fixed material due to smaller fragment size. Nevertheless, this fragment is sufficient for chip hybridization. Amplifiable DNA till 381 bp was detected in historical long-term preserved tissues fixed for more than 80 years [27].

We detected HPV type 16 in all four genital carcinomas which supporting the present belief that HPV is a major etiologic factor in anogenital cancer for both female and male patients [33]. Interestingly, in the specimen wGt151 the histological and molecular genetic results mismatched as it showed no cancer-like histology but evidence of HPV-DNA. Since no other pathology could be confirmed microscopically, it is most likely that we did not biopsied a representative sample of the specimen. However, the macroscopic appearance of the specimen matches with the original diagnosis of cervical cancer. HPV type 16 does not necessarily turn every infected epithelial cell into a cancer cell [34]. Therefore, non-dysplastic epithelial cells can be positive for HPV type 16, especially in patients with cervical cancer. Interestingly, HPV DNA was also present in 4/7 (57%) head and neck carcinomas (HNC). This is in contrast with the assumption that HPV accounted for only a small proportion of HNC development in former populations compared to other risk factors such as alcohol or tobacco consumption (reviewed in [35]). This statement was mainly made due to the increased smoking rate from the beginning of the 20^th^-century [36,37] as well as different sexual behaviour with less practice of oral sex [38,39]. So far, no reliable data about the HPV prevalence in HNC before 1970 is available since HPV was first discovered in the 1970s [40]. However, Näsman et al. found the incidence of HPV-positive cancers to have increased from 23.3% in the 1970s to 93% in 2006 / 2007 in Sweden [41]. Therefore, an HPV prevalence of more than 50% in our HNC preparations from the first half of the 20^th^ century is notable. However, all of our HNC cases were located at oro-pharyngeal and not at deeper laryngeal or hypo-pharyngeal regions. These sites are naturally more vulnerable to HPV infection [42] and therefore show a significantly higher HPV prevalence [43]. Due to the relatively small number of HNC cases in this study, we cannot make a definitive statement about the prevalence of HPV in HNC between 1900 and 1960, but our results would encourage conducting further research on this topic.

We detected mycobacterial DNA in 13/14 TB specimens. In four specimens (Lu124, Lu145, Lu217 and Gf122) we found DNA of the MTB complex that includes different pathogens such as *M*. *tuberculosis* or *M*. *bovis*. During the 1930s, when many of our samples originate, agriculture was an integral part of North-East German society with approximately 50% of the population employed in agriculture. 63% of bovine herds were infected with tuberculosis due to *M*. *bovis* infection at this time [44]. Bovine tuberculosis was a major source of infection mainly in children (up to 50%), due to consumption of raw milk or air-borne infection, whereas outbreaks of this disease at an older age occurred after any weakening of the immune system [44–46]. Hence, it is possible that the detected tuberculosis infection in the four specimens was due to exposure to infected bovine herds. For the differentiation between *M*. *bovis* and other species of the MTB complex, the sequence of the *pncA* gene was determined. The mutation in question could not be detected in any of the four respective specimens and thus, *M*. *bovis* could be excluded. It must be mentioned, that the exclusion of *M*. *bovis* is not a full proof of *M*. *tuberculosis*. Nevertheless, taking into account that *M*. *tuberculosis* is the most common cause of TB and all four preparations showed typically macroscopic and microscopic patterns of TB, *M*. *tuberculosis* is the most likely pathogen. However, it is surprising that we only detected *M*. *tuberculosis* in approximately 20% of our TB preparations. In contrast, the majority of our preparations were positive for the MOTT complex that consists of more than 120 pathogenic species [47]. The two specimens Lu145 and Gf122 showed a co-infection with MTB and MOTT which seems to be common in TB patients. For example, in a group of patients with pulmonary TB presented by Damaraju et al., 11% were co-infected with other mycobacteria [48]. However, it is notable that more than half of our specimens were only positive for MOTT but not for MTB.

Our results are encouraging but limitations of the study should be kept in mind. First, the possibility of false results due to severe DNA degradation should be kept to a minimum. Predominately, DNA degradation leads to G>A substitutions due to deamination of cytosine and transformation into uracil on the complementary strand [49]. It is possible that wrong DNA sequences, created by replication of degraded DNA, imitate the sequence of HPV or mycobacterial DNA and cause false-positive hybridization signals. However, since these events happen by chance, it is very unlikely that the same nucleotide positions are affected in a repeated replication. To strengthen the significance of our results, we repeated all PCR and hybridization analyses obtaining identical results in each round. Furthermore, we treated the DNA of several specimens with UDG prior to the amplification step. This UDG treatment destroys all DNA strands harbouring uracil due to degradation and thus, minimizes errors caused by degraded DNA dramatically [50]. Therefore, we consider our hybridization results as true. Second, pathological collections often do not reflect the true incidence of certain diseases in these former times. There is always a collection bias, for example, when different organs were derived from only one patient. Since no data is available from the corresponding patients, their clinical histories or even fixation dates of the specimens, we cannot rule out that, for example, all our TB specimens belonged to only two or three patients. Finally, the issue of contamination is problematic when working with long-term preserved specimens. Although all efforts have been made to avoid contamination in the laboratory (e.g. using facilities designed for molecular genetic work, sterile hoods, wearing gloves etc.), it is hard to exclude that contamination occurred prior to the handling in this study. Thus, it cannot be ruled out that we detected false-positive signals due to pre-laboratory contamination and all cases should be validated in a synopsis of all histological and molecular genetic results. However, the fact that we only found the cancerogenic HPV type 16 and no other HPV types in our cancer specimens makes contamination less likely since contamination happens by chance. The same argument applies to the TB specimens in which only MTB was found. Nevertheless, the surprisingly high number of MOTT positive cases in our collection needs to be discussed against the background of both contamination and collection bias. The possibility that our MOTT-positive specimens are derived from only a few patients could well explain why MOTT associated diseases are over-represented in our collection, as compared to the general patient population. However, pre-laboratory contamination is also a possible explanation for our observations, especially when considering two facts: (1) Three MOTT-positive specimens (Kn322, Lu30/10 and wGT200) are lacking any histological evidence for a mycobacterial disease and (2) a sole infection with MOTT does not inevitably proof that the patient suffered from a MOTT caused disease. Indeed, western laboratories nowadays detect MOTT more often than MTB. However, only 50% of these cases present clinically with a real MOTT disease, whereas contamination should be always considered in the remaining cases [51]. Furthermore, again, this highlights the importance of a multi-modal approach for evaluating archival specimens. Taken together the histological and molecular genetic data, we think our positive MOTT results are most likely due to pre-laboratory contamination. Further investigations such as DNA sequencing may help to address this issue since typical DNA degradation pattern occurs after a certain amount of time and may not be detectable in younger DNA contamination [52]. However, it is almost impossible to distinguish true signals against contamination in archival material when the contamination happened decades ago since both would show the same pattern of fragmentation and degradation in this case [53].

In summary, our study shows that long-term preserved tissue from medical collections can be used in university teaching and research since the microscopic structures are often well preserved and even molecular genetic investigations are feasible. Moreover, our study demonstrated that investigation of historical specimens can yield interesting results. However, working with old pathological material requires caution. First, the original diagnosis should always be questioned. Second, the amount of tissue available is limited so that the material should be handled with care. Finally, the risk of collection bias needs always to be kept in mind.
